# Oxidative Degradation of High-Molar-Mass Hyaluronan: Effects of Some Indole Derivatives to Hyaluronan Decay

**DOI:** 10.3390/ijms21165609

**Published:** 2020-08-05

**Authors:** Katarína Valachová, Mojmír Mach, Ladislav Šoltés

**Affiliations:** Centre of Experimental Medicine of the Slovak Academy of Sciences, Institute of Experimental Pharmacology & Toxicology, Dúbravská cesta 9, SK-84104 Bratislava, Slovakia; katarina.valachova@savba.sk (K.V.); ladislav.soltes@savba.sk (L.Š.)

**Keywords:** high-molar-mass biopolymer, indole derivatives, isatin, radical scavenging capacity, reactive oxygen species, rotational viscometry

## Abstract

Indole derivatives such as isatin (a natural compound), cemtirestat, stobadine, and its derivatives (synthetic compounds) are known to have numerous positive effects on human health due to regulation of oxidative status. The aim of the study was to assess radical scavenging capacities of these compounds and explore their potential protective effects against reactive oxygen species formed during Cu(II) ions and ascorbate-induced degradation of high-molar-mass hyaluronan. Based on the IC_50_ values determined by the ABTS assay, the most effective compound was SM1M3EC2·HCl reaching the value ≈ 11 µmol/L. The lowest IC_50_ value reached in the DPPH assay was reported for cemtirestat ≈ 3 µmol/L. Great potency of inhibition of hyaluronan degradation was shown by cemtirestat, followed by isatin even at low concentration 10 µmol/L. On the other hand, stobadine·2HCl had also a protective effect on hyaluronan degradation, however at greater concentrations compared to cemtirestat or isatin. SME1i-ProC2·HCl reported to be a less effective compound and SM1M3EC2·HCl can be considered almost ineffective compared to stobadine·2HCl. In conclusion, our results showed that both isatin and cemtirestat were capable of attenuating the degradation of high-molar-mass hyaluronan due to their ability to complex/sequester cupric ions.

## 1. Introduction

Oxidation simply means chemical reaction of a substance with a molecule of dioxygen (O_2_), yet reduction is generally not liberation of O_2_, e.g., when the substance decomposes. When the substance is oxidized (in aqueous media) we may claim that a transfer of an electron (e^−^_aq_) occurs, or more precisely one e^−^_aq_ transfers from the parent substance to the electron acceptor. Vice versa, during the reduction reaction one e^−^_aq_ is trapped by the electron accepting substance.

Several simple chemical assays to study the electron/proton donating (accepting) properties of various substances have been established. The photometric ABTS and DPPH assays are represented below:

ABTS^•+^ + electron donating substance → ABTS + reduced substance (a free radical) (1)

DPP^•^ + H donating substance → DPPH + reduced substance (a free radical) (2)

While the ABTS assay runs in aqueous solutions where the e^−^_aq_ may freely transfer, the DPPH assay, due to the 2,2-diphenyl-1-picrylhydrazyl radical limited solubility in aqueous media, has been performed in an aliphatic alcohol. Both assays are still very popular and the determined values (e.g., IC_50_) are frequently exploited to classify the investigated substance as more or less effective. However, one should take into consideration that the output of the measurements indicates not the “true antioxidative property” but just an apparent value of the electron/proton donating (accepting) properties of the assayed substance under the applied experimental conditions.

In accord with “the pecking order of free radicals and antioxidants”, reviewed by Buettner [1], free radicals vary widely in their thermodynamic properties, ranging from very oxidizing to very reducing. Thus, one-electron reduction potential of free radicals (classified also as the pecking order of free radicals) is in agreement with experimentally observed free radical electron (hydrogen atom) transfer reactions (cf. Table 1).

The positive value of standard one-electron reduction potential (E°′) of free radicals indicates the radical oxidizing properties, while negative E°′ values mean the free radical reducing potential. It is thus self-explanatory that, e.g., free hydroxyl radical (HO^•^), by accepting one electron in the presence of H^+^ (H_3_O^+^ in aqueous medium), turns to water molecule at 2.31 V. So, the free ^•^OH radical in aqueous media is represented by the highest affinity to trap (H^+^ + e^−^_aq_), i.e., one hydrogen radical (H^•^). In other words, the free hydroxyl radical in contact with a biologically important compound by abstracting one hydrogen radical (hydrogen atom) turns into molecule of water and usually yields a destructed bio-structure.

Informed readers of this communication know that not only free radicals (HO^•^, RO^•^, ROO^•^, ArO^•^, ArOO^•^) but also not charged/neutral molecule (H_2_O_2_) and superoxide anion radical (O_2_^•−^) have been ranked among reactive oxygen species (ROS). It should be however pointed out here that ROS in the living body have inevitable functions so their presence in physiologically optimal concentration is regulated by numerous, mostly enzyme-driven reactions.

Based on the data given in Table 1, one should primarily focus on methods designed to generation of HO^•^, aliphatic alkoxyl and peroxyl radicals (RO^•^ and ROO^•^). From the point of view of free-radical reactions, the primary choice on working with RO^•^ and/or ROO^•^ type radicals leads usually to peroxidation of a lipid. As the physiologically relevant source of ^•^OH radicals one might choose the Fenton reaction (Fe(II) + H_2_O_2_ → ^•^OH + HO^−^ + Fe(III)). However, since ferrous ions are susceptible to oxidation, Fe(II) ions can be replaced with cupric ones. Along with this replacement, hydrogen peroxide is substituted by ascorbate, which is omnipresent within the human organism. By these two replacements we work with the so-called Weissberger’s biogenic oxidative system (WBOS): Under aerobic conditions equimolar concentrations of mono-anion of ascorbate (AscH^–^) and cupric ions, through intermediate Cu(I)—complexes, yield hydrogen peroxide, which decomposes to hydroxyl radicals recovering Cu(II) ions (Valachová et al. (2016) [2]).

**Scheme 1:**
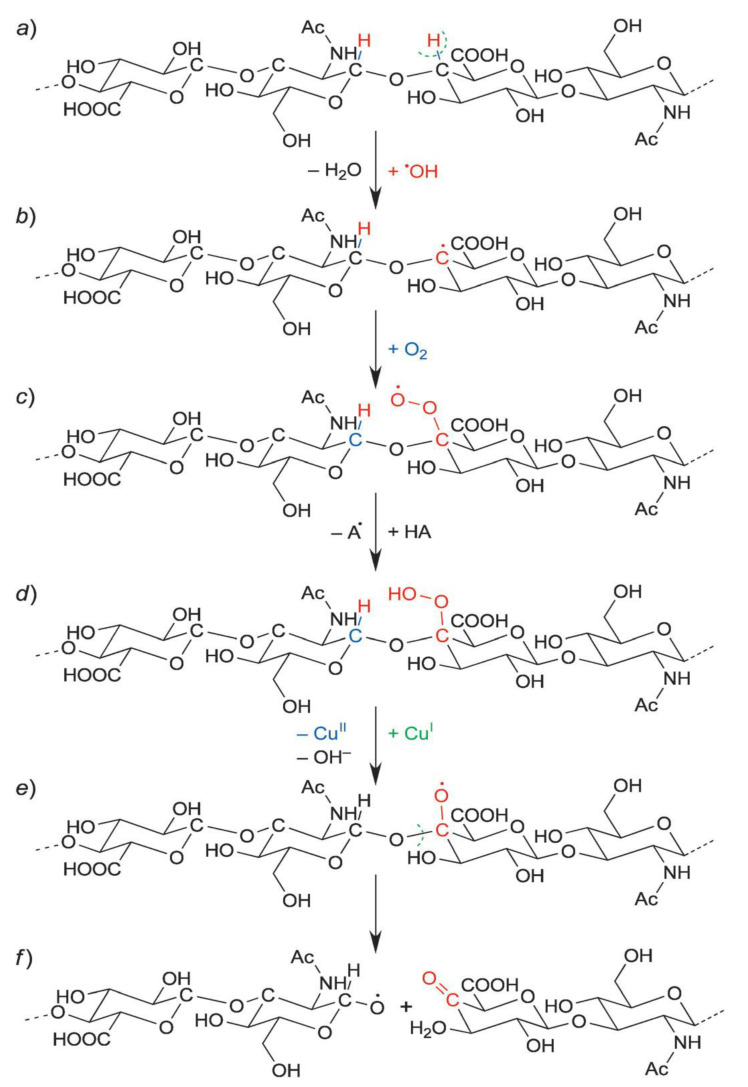
Reaction of initiation: (**a**) An intact HA macromolecule reacts with ^•^OH radical; (**b**) formation of an intermediate, i.e., a *C*-centered HA macroradical (denoted later A^•^). Reactions of propagation and of transfer of free-radical centre: (**c**) formation of peroxy-type macroradical; (**d**) and (**e**) generation of HA hydroperoxide and a highly unstable alkoxy-type macroradical. The reaction yielding fragments: (**f**) alkoxy-type macroradical and HA macromolecule bearing a terminal C=O group. Both fragments are represented by reduced molar masses. (Hrabárová et al. (2012) [3]).

Hydroxyl radicals—one of the most reactive species—are responsible for initiation and the subsequent perpetual propagation of free-radical peroxidation of the lipid. One criticism on employing the lipid (or more often its salt) may potentially be the fact that the microenvironment surrounding the lipid molecule in the organism consists of hydrophobic solubilizers. Thus, the prerequisite of free transfer of e^−^_aq_ (and/or H^•^) is most plausibly not fulfilled. To study the perpetual peroxidation reaction in aqueous media, instead of any lipid we advocate exploiting an endogenous macromolecule, namely high-molar-mass hyaluronan (HA) [3]. Under such experimental conditions the sequence depicted on Scheme 1 of (radical) chain reactions can occur.

**Figure 1:**
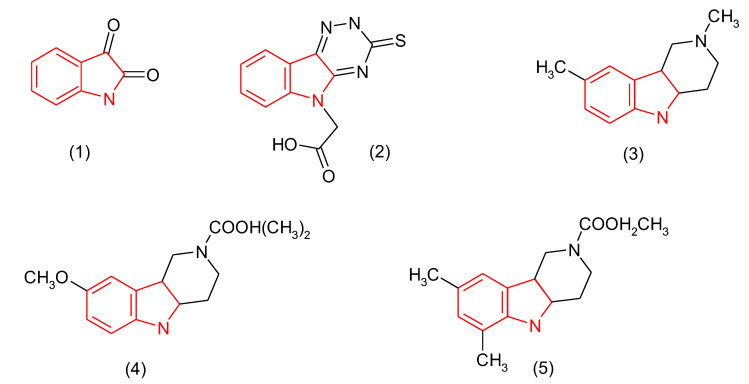
Isatin (**1**), cemtirestat (**2**), stobadine (**3**), SME1i-ProC2 (**4**), and SM1M3EC2 (**5**).

Isatin—1H-indole-2,3-dione ((**1**) in Figure 1)—is a heterocyclic compound, present in many plants and human organism [4,5]. Isatin possess antimicrobial, anti-inflammatory, antitumor, anticonvulsant, anthelmintic, analgesic, antiviral, anti-HIV, and antioxidative actions. Cemtirestat—2-(3-thioxo-2*H*-[1,2,4]triazino[5,6-b]indole-5(3*H*)-yl)acetic acid [(**2**) in Figure 1]—which can be prepared by a three-step synthesis from isatin [6] is classified as a highly selective and efficient aldose reductase inhibitor with antioxidant properties [7,8].

Štolc et al. [9] introduced a synthetic indole compound stobadine—(–)-cis-2,8-dimethyl- -2,3,4,4a,5,9b-hexahydro-1*H*-pyrido[4,3b]indole ((**3**) in Figure 1)—which can be classified as an optically active form of the clinically used racemic drug carbidine [10]. Stobadine, along with cardioprotective, neuroprotective, antihistamine, and antiulcerous effects, demonstrates antioxidative and free-radical scavenging properties [11,12,13,14,15,16,17,18,19,20].

When visually inspecting the structural formulae of compounds (**1**), (**2**), and (**3**) it is obvious that the (indolic) nitrogen atom in both—isatin and stobadine—bears an atom H, while in cemtirestat the hydrogen atom is replaced with the acetyl group. And since Steenken et al., even in 1992 [21], reported on the formation of an indolic nitrogen-centered radical of stobadine in reaction with various free radicals including ^•^OH, ROO^•^, and ArO^•^, we focused on employing the compounds (**1**), (**2**), and (**3**) along with two stobadine derivatives ((**4**) and (**5**) in Figure 1) to function as preventive and chain-breaking scavengers of perpetual free-radical degradation of high-molar-mass hyaluronan. The poor water solubility of the basic compound (**3**) and those of (**4**) and (**5**) was circumventing by preparation of dihydro- or monohydrochloride salt forms.

Thus, the aim of this study is to explore the effects of five indole derivatives ((**1**)–(**5**)) on oxidatively degraded high-molar-mass hyaluronan by Weissberger’s biogenic oxidative system. The ABTS and DPPH assays were applied for in more detail understanding the observations when working with the WBOS experimental arrangement.

## 2. Results

Table 2 and Table 3 summarize the IC_50_ values of isatin, cemtirestat, stobadine·2HCl, SME1i-ProC2·HCl, and SM1M3EC2·HCl determined by both ABTS and DPPH assays. Results of the ABTS assay (Table 2) show that the order of IC_50_ values (in µmol/L) is 5 < 3 < 2 < 4. As expected, the order of IC_50_ values determined by the DPPH assay (Table 3) led to a different order: 2 < 4 < 5 < 3 < 1 (in µmol/L).

Both ABTS and DPPH assays are useable, however, the output of the measurements relates not to the true antioxidative property of the given compound but just to an apparent value of the “reductive property” of the compound assayed under the used experimental conditions.

**Figure 2:**
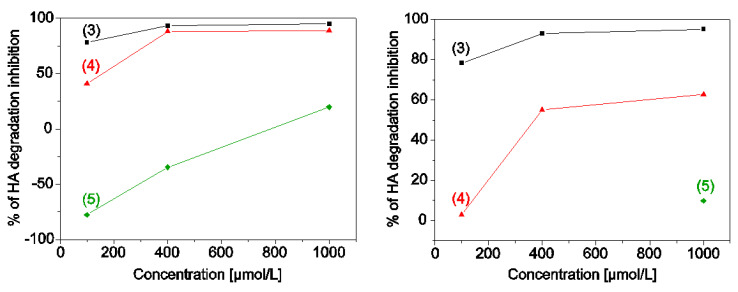
Percentage of inhibition of HA degradation within 120 min. Stobadine⋅2HCl dihydrochloride, i.e., compound (**3**), and two of its derivatives ((**4**) and (**5**)) were examined as preventive antioxidants (left panel) and chain-breaking antioxidants (right panel).

Figure 2, left panel, illustrates the results of percentage of inhibition by the compound (**3**), (**4**), or (**5**) at the concentration range 100–1000 µmol/L within 120 min in the experimental design (a) (cf. Section “Study of uninhibited/inhibited hyaluronan degradation”) where the preventive inhibitory action of the compound was studied. As could be estimated from Figure 2, left panel, the compound (**3**) demonstrated a high protective effect even at the lowest concentration (100 µmol/L). On contrary, the compound (**5**) pronounced significantly HA degradation effect during 120 min. The compound explored at the highest concentration (1000 µmol/L) was shown to have just a minor protection against HA degradation (cf. Figure 2, left panel).

When applying the experimental design (b) (cf. Section “Study of uninhibited/inhibited HA degradation”) the compound (**3**) was the most potent to act as the chain-breaking antioxidant. There are several papers documenting that stobadine has been postulated as the chain-breaking antioxidant able to scavenge chain-propagating peroxyl radicals [13,20,21,22,25].

However, although the compound (**5**) at the highest concentration was shown to have a slight protective effect (cf. Figure 2, both panels), this compound could be classified as inefficient.

**Figure 3:**
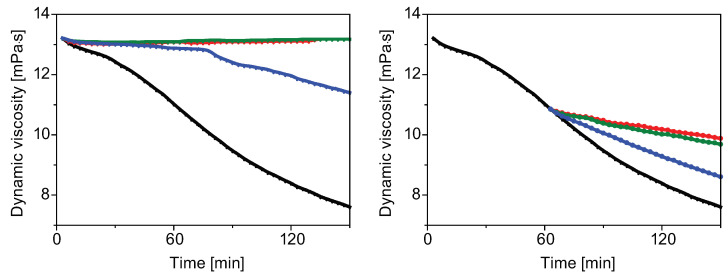
Time-dependent changes in dynamic viscosity of HA solutions exposed to oxidative degradation initiated by Cu(II) ions and ascorbate (black curve). Cemtirestat was added to the reaction mixture before HA degradation begins (left panel) or 1 h later (right panel) at micromolar concentrations: 25 (red), 10 (green) and 1 (blue).

Results in Figure 3, left panel, display that cemtirestat completely inhibited the ^•^OH radical-induced HA degradation even at concentrations 25 and 10 µmol/L (red and green curves). Moreover, even at the lowest concentration (1 µmol/L; blue curve) cemtirestat was still in part a potent antioxidant. As evident from the results in Figure 3, right panel, cemtirestat at all three assayed concentrations showed a significant inhibition of the chain-breaking period of HA decay (cf. Scheme 1, the reaction steps from (c) to (d) and from (e) to (f). The later observation is supported also by results of Šoltésová Prnová et al. [26].

**Figure 4:**
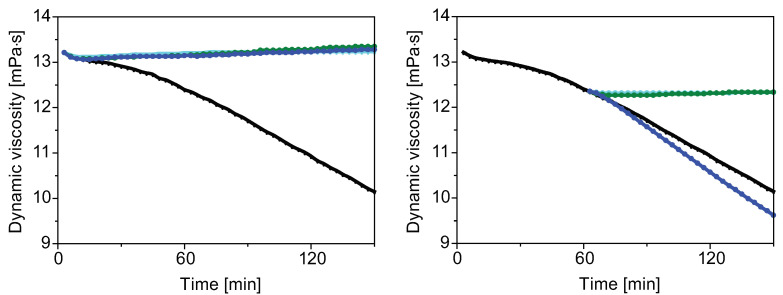
Time-dependent changes in dynamic viscosity of HA solutions exposed to oxidative degradation initiated by Cu(II) ions and ascorbate (black curve). Isatin was added to the HA reaction mixture before HA degradation begins (left panel) and 1 h later (right panel) at micromolar concentrations: 100 (cyan), 10 (green) and 1 (blue).

As indicative from the results in Figure 4, right panel, isatin at concentrations 10 and 100 µmol/L completely inhibited the chain-breaking period of HA decay (cf. Scheme 1, the reaction steps from (c) to (d) and from (e) to (f)). Isatin at its lowest level (1 µmol/L) was inefficient and moreover it minutely enhanced the degradation of HA macromolecules.

## 3. Discussion

The ABTS cation radical (ABTS^•+^) is reactive towards most electron donors (antioxidants). During the reaction (1), the blue green color of ABTS cation radical is converted to a colorless substance. Two limitations should however be mentioned right here: (i) Since the center of a cation radical is “localized within nitrogen atom”, the electronegativity of ABTS^•+^ would be in some cases weaker than the electronegativity of the counterpart atom in the investigated compound; (ii) due to the fact that the decolorization reaction (1) is monitored photometrically, some colorful compounds themselves disable the exact measurements. Thus, due to these two limitations, the ABTS assay in some cases does not yield any IC_50_ value and one should claim that the parameter investigated is undeterminable (cf. Table 2, compound isatin). When applying the DPPH assay, the results are similar, which was demonstrated by Šekularac et al. [27], who found very slight, if any activity of isatin against DPP^•^ radical. The centre of radical in DPP^•^ probe is localized mostly within nitrogen atom [28] and the intense purple color of DPP^•^ radical on abstracting an atom of hydrogen (H^•^) alters to yellow [29].

Although the IC_50_ values listed in Table 2 represent not “the true antioxidative property” of the compounds investigated but just an apparent value of reductive property of the assayed molecule under the applied experimental conditions, the parameters determined by the ABTS assay are useable: The informed reader can guess the potential circumstances on redox reactions among the compound (**2**), (**3**), (**4**) or (**5**) when it comes to contact with either Cu(I)—complex or mono-anion of ascorbate—AscH^–^.

The parameters listed in Table 3 summarize the IC_50_ values of isatin, cemtirestat, stobadine·2HCl, SME1i-ProC2·HCl, and SM1M3EC2·HCl obtained by the DPPH assay. By comparing the values just for stobadine·2HCl and its two structural isomers, one could predict that due to the order 4 < 5 <<< 3, the molecule of stobadine·2HCl will be at least an efficient donor of hydrogen atom. Similarly, Zalibera et al. [30] showed for SM1M3EC2·HCl greater trolox equivalent of antioxidant capacity (TEAC) determined by DPPH assay compared to stobadine·2HCl.

However, as documented by results represented in Figure 2, stobadine is the most potent compound either against the initiation reaction compared to its two derivatives during high-molar-mass hyaluronan oxidative degradation (cf. left panel in Figure 2) or against the chain-breaking propagation reaction during free-radical HA decay. Although the molar amount of the applied compound (**3**), (**4**), or (**5**) exceeded the concentration of ascorbate, stobadine·2HCl molecules and that of SME1i-ProC2·HCl at greater levels 400 or 1000 μmol/L may be claimed as efficient enough to cease the reaction of initiation (cf. Scheme 1, reaction steps from (a) to (b)). Yet, surprisingly the SM1M3EC2·HCl molecules do not function protectively against the initiation reaction during high-molar-mass HA oxidative degradation (cf. left panel in Figure 2) but, on contrary, the compound (**5**) was a significant promotor of free-radical HA decay initiated under the WBOS conditions. Simultaneously, by comparing the experimental results represented in Figure 2, right panel, exclusively the molecules of stobadine·2HCl at 400 or greater level 1000 μmol/L were reported to interrupt perpetual propagation of HA free-radical degradation ((cf. Scheme 1, reaction steps from (c) to (d) as well as from (e) to (f)).

According to the IC_50_ value determined for cemtirestat ≈ 3 µmol/L, one could state that this drug is approx. 40-times more effective H atom donor than stobadine·2HCl (IC_50_ ≈ 120 µmol/L; cf. Table 3). Thus, the observed total inhibition of the ^•^OH radical-induced HA degradation even at cemtirestat concentrations 25 and 10 µmol/L (Figure 3, left panel) looks self-explanatory. Yet the deeper insight into the high inhibitory action of cemtirestat results to another thesis: this drug under the used experimental condition could be present in two tautomeric forms, namely that of chemical structure represented in Figure 1 compound (**3**) as well as 2-(3-mercapto-5*H*-[1,2,4]triazino [5–6-b]indole-5-yl)acetic acid):


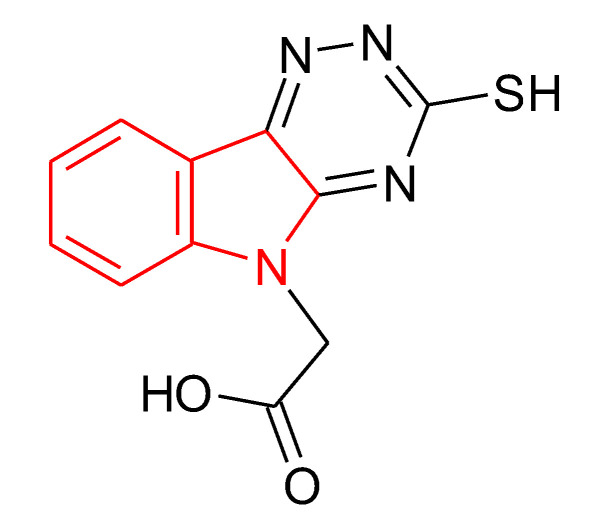


The above mentioned cemtirestat tautomer in general is a compound bearing a mercapto/thiol functional group. Since thiol groups may efficiently trap/complex cupric ions (Ar-S-Cu-S-Ar) no “free/catalytically active” Cu(II) ions have been available for the WBOS reaction. The later tenet is indirectly supported by the observation shown in Figure 3, left panel: When just 1 µmol/L cemtirestat was applied expecting the total inhibition of the ^•^OH radical-induced HA degradation, the observed result can be explained as follows: 2 µmol cemtirestat would be necessary to add to 1 µmol Cu(II) ions to accomplish the total copper ions trapping (Ar-SH:Cu(II) = 2:1). A really very interesting result can be observed in Figure 3, right panel, since during 1 h the reactions described in WBOS and Scheme 1 run “uninhibitedly”. At the beginning of application of cemtirestat one should admit that the reaction vessel contains HA hydroperoxides A-OOH in accord with step (d) in Scheme 1. The addition of cemtirestat at any assessed level (1–25 μmol/L) should reduce cupric ions (1 µmol/L) to those of Cu(I) ones, and thus the reaction should result in fast decomposition of A-OOHs in accordance with:

A-OOH + Cu(I) + 2H^+^ → A-OH + Cu(II) + H_2_O (3)

The decomposition of a fraction of HA hydroperoxides results in inhibition of the propagation of HA degradation since the reaction step (f) from (d) (cf. Scheme 1) is partly interrupted.

Results in Figure 4, left panel, support the statement that isatin within the whole concentration range applied completely inhibited the ^•^OH radical-induced HA degradation. Since isatin H atom donoring capacity can be classified as very weak—IC_50_ ≈ 200 µmol/L—the informed reader according to Venkata Ramana Reddy and Ram Reddy [31] will accept the tenet that 1:1 or 1:2 complexes are formed between cupric ions and either isatin or its tautomer—2-hydroxy-indole-3-one:


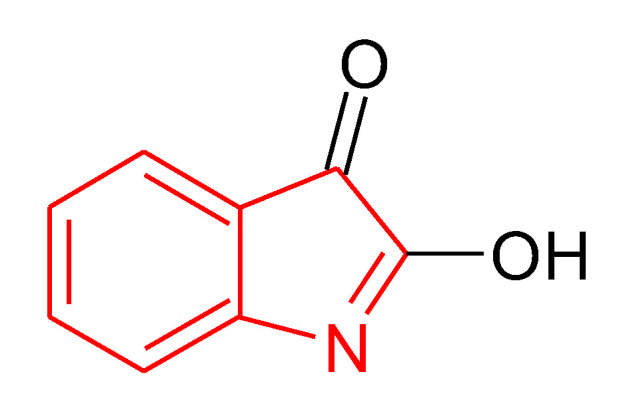


By that way, no catalytically active cupric ions are present to initiate the hydrogen peroxide generation. Simultaneously, when comparing the reaction steps from (d) to (e) and (f) in Scheme 1, it is obvious that complexing/trapping copper ions by isatin, no A-OOHs fragmentation will carry out. This statement is supported by results shown in Figure 4, right panel at the two greater isatin concentrations (100 and 10 µmol/L). A slight HA degradation promotion at 1 µmol/L isatin concentration was reported, however no straight explanation may be pointed out. A simple speculative tenet is by “loosing” copper ions due to their complexing by isatin, no charge to physico-chemical “crosslinking” between HA polyanions and metal cations may more occur, whose decrement may result in the given facilitated declination of the monitored dynamic viscosity value (cf. Figure 4, right panel).

By advocating the above mentioned study relevance, one should take into account that: HA is a linear polysaccharide composed of two alternating units, β-1,4-d-glucuronic acid and β-1,3-*N*-acetyl-d-glucosamine. This biopolymer is extruded from special cells in molar size equaling up to several millions of Daltons. HAs in vertebrates, associated with proteins, are structural components of extracellular matrix, skin, joints, and cornea. Macromolecules of HA make a specific “tissue”—synovial fluid—which function not only as a lubricant of bone cartilages but also as a highly efficient antioxidant. HA oxidative degradation within synovial fluid maintains this tissue hypoxic, i.e., physiologically optimal for cartilage chondrocytes [32]. As patented [33], the method involved under the presented study, brought novel/relevant data on free-radical scavenging properties of the compound ((**1**), (**2**), (**3**), (**4**), and (**5**)), i.e., how it functions either as the protective or the chain-breaking antioxidant.

## 4. Materials and Methods

### 4.1. Materials

Hyaluronan sample of high molar mass (M_w_ = 1.53 MDa; M_w_/M_n_ = 1.76) was purchased from Lifecore Biomedical Inc., Chaska, MN, USA. l-Ascorbic acid and K_2_S_2_O_8_ p.a. were the products of Merck KGaA, Darmstadt, Germany. NaCl and CuCl_2_·2H_2_O p.a. were purchased from Slavus Ltd., Bratislava, Slovakia. 2,2′-Azino-bis(3-ethylbenzothiazoline-6-sulfonic acid) diammonium salt (ABTS; purum, >99%) was from Fluka, Steinheim, Germany. 2,2-Diphenyl-1-picrylhydrazyl radical (DPP^•^) and methanol were purchased from Sigma-Aldrich, Steinheim, Germany. Deionized water of high-purity (conductivity of ≤0.055 µS/cm), was made by using the TKA water purification system (Water Purification Systems GmbH, Niederelbert, Germany). Isatin was purchased from Sigma-Aldrich, Prague, Czech Republic. Cemtirestat was a product of Akos Consulting & Solutions, Wurttemberg, Germany. Stobadine·2HCl, SM1M3EC2·HCl, and SME1i-ProC2·HCl (cf. Figure 1) were prepared at the Institute of Experimental Pharmacology and Toxicology, Bratislava, Slovakia.

### 4.2. Preparation of Stock and Working Solutions

The HA sample (2 mg/mL) was dissolved overnight in 0.15 mol/L aqueous NaCl in two steps: At first, 4.0 mL of the solvent was added to 16 mg HA, after 6 h 3.9 or 3.85 mL of 0.15 mol/L NaCl was added. The stock solutions of l-ascorbic acid (16 mmol/L), the stock solutions of compounds (**1**) (16 mmol/L) or (**2**) (3.8 mmol/L) and stock solution of cupric chloride (160 µmol/L) were also prepared in 0.15 mol/L aqueous NaCl.

### 4.3. Study of Uninhibited/Inhibited Hyaluronan Degradation

Firstly, HA degradation was induced by the oxidative system composed of CuCl_2_ (1 µmol/L) and l-ascorbic acid (100 µmol/L). The procedure was as followed: CuCl_2_ stock solution in volume 50 µL was added to the HA solution (7.90 mL), stirred for 30 s and the reaction mixture was left to stand for 7 min 30 s at room temperature. Then, 50 µL of stock l-ascorbic acid solution was added to the HA solution and stirred again for 30 s. The solution mixture was then immediately transferred into the viscometer Teflon^®^ cup reservoir.

The procedures to examine the compound (**1**) or (**2**) as an inhibitor of HA degradation were as followed:

(a) The stock solution of CuCl_2_ in the volume of 50 µL was added to the HA solution (7.85 mL), which after stirring for 30 s was left to stand for 7 min 30 s at room temperature. Then, 50 µL of the appropriately diluted stock solution of compound (**1**) or (**2**) was added and the solution was stirred again for 30 s. Finally, 50 µL of stock l-ascorbic acid solution was added and the completed solution was stirred for 30 s. The solution mixture was then immediately transferred into the viscometer Teflon^®^ cup reservoir.
(b) In the second experimental setting a similar procedure as that described in (a) was applied, however, after the reaction mixture standing for 7 min 30 s at room temperature, 50 µL of stock l-ascorbic acid solution was added. After 1-h stirring of the reaction mixture, finally 50 µL of the appropriately diluted stock solution of the compound (**1**) or (**2**) was added and stirred again for 30 s. The reaction mixture was then immediately transferred into the viscometer Teflon^®^ cup reservoir.

The measurement of changes in the dynamic viscosity value was carried out for 2.5 h at temperature 25 °C. The viscometer Teflon^®^ spindle rotated at 180 rpm, i.e., at a shear rate of 237 s^−1^. The viscosity data were reported in 3-min intervals by using rotational viscometer (Brookfield Engineering Labs, Inc., Middleboro, MA, USA).

The measurements of protective/promoting actions of indole derivatives (**3**), (**4**), and (**5**) against high-molar-mass hyaluronan oxidative degradation were performed analogously to the above study design [34,35]. The stock solutions of compounds (**3**), (**4**), and (**5**) were 160 mmol/L.

### 4.4. ABTS and DPPH Assays—Determination of IC_50_ Values

The ABTS^•+^ concentrated solution, formed by reaction of potassium persulfate (3.3 mg dissolved in 5 mL of deionized water) with ABTS (17.2 mg), was maintained overnight below 0 °C. Before carrying out the experiment, the ABTS^•+^ concentrated solution (1 mL) was diluted with deionized water (60 mL). The working ABTS^•+^ solution (250 µL) was added to 2.5 µL of the solution of compound (**1**, **2**, **3**, **4**, or **5**) at the concentration range 8−250 µmol/L and after 6 min the absorbance was measured at 734 nm.

The working DPP^•^ solution was formed by dissolving the 2,2-diphenyl-1-picrylhydrazyl radical (1.1 mg) in methanol. The DPP^•^ solution (25 µL) at the concentration range 2−1000 µmol/L was added to 225 µL of the solution of the examined compound (**1**, **2**, **3**, **4**, or **5**). The absorbance was measured at 517 nm after 30 min.

The photometric measurements were performed quadruplicately in 96-well Greiner UV-Star microplates (Greiner-Bio-One GmbH, Germany) with Tecan Infinite M 200 reader (Tecan AG, Austria). The IC_50_ values were calculated with CompuSyn 1.0.1 software (ComboSyn, Inc., Paramus, HJ, USA).

## 5. Conclusions

We confirmed the reports on stobadine·2HCl to be potent in scavenging ^•^OH, alkoxy-, and peroxy-type radicals. As a method we selected rotational viscometry and as a marker the damage of high-molar-mass hyaluronan. We found out that the derivative SME1i-ProC2·HCl was shown to be less potent in attenuating hyaluronan degradation and SM1M3EC2·HCl can be considered ineffective compared to stobadine·2HCl. Much more potent indole derivatives examined in oxidatively degraded hyaluronan were isatin and cemtirestat, which were effective in much lower concentrations to reach total inhibition of hyaluronan degradation than stobadine·2HCl and its derivatives. Such an effect is suggested to be due to complexation/sequestering of cupric ions.

Concerning to results of both ABTS and DPPH assays (expressed in µmol/L), the highest radical scavenging capacities were reported for SM1M3EC2·HCl and cemtirestat, respectively. On contrary, the IC_50_ value in the ABTS assay for isatin was impossible to determine and in the DPPH assay this value was rather high.

## Figures and Tables

**Table 1:** Redox couple (one-electron reductions) [1].

Redox Couple ^a^	E°′/mV
HO^•^, H^+^/H_2_O	+ 2310
RO^•^, H^+^/ROH (aliphatic alkoxyl radical)	+ 1600
ROO^•^, H^+^/ROOH (aliphatic peroxyl radical)	+ 1000
GS^•^/GS^−^ (glutathione)	+ 920
TO^•^, H^+^/TOH (tocopherol)	+ 480
H_2_O_2_, H^+^/H_2_O, HO^•^	+ 320
Asc^•−^, H^+^/AscH^−^ (ascorbate)	+ 282
O_2_/ O_2_^•−^	− 160
RSSR/ RSSR^•−^ (e.g., oxidized glutathione)	− 1500
H_2_O/e^−^_aq_	− 2870

^a^ The listed redox couples are selected from those free radical reactions that are of interest to the medicinal sciences.

**Table 2:** Values of IC_50_ determined by ABTS assay.

Compound	IC_50_ [µmol/L]
1. Isatin ^a^	undeterminable
2. Cemtirestat	26 ± 1.8
3. Stobadine·2HCl ^b^	12.6 ± 0.24
4. SME1i-ProC2·HCl ^c^	27.6 ± 0.46
5. SM1M3EC2·HCl ^d^	10.8 ± 0.38

The IC_50_ values are means ± SEMs. The IC_50_ value of isatin ^a^ was not determinable [22]. The IC_50_ values of stobadine·2HCl ^b^, SME1i-ProC2·HCl ^c^ and SM1M3EC2·HCl ^d^ were determined by Valachova et al. (2014) [23].

**Table 3:** Values of IC_50_ determined by DPPH assay.

Compound	IC_50_ [µmol/L]
1. Isatin ^a^	203
2. Cemtirestat	2.9 ± 0.3
3. Stobadine·2HCl ^b^	122 ± 5
4. SME1i-ProC2·HCl ^c^	10.6 ± 0.5
5. SM1M3EC2·HCl ^d^	21 ± 1.4

The IC_50_ values are means ± SEMs. ^a^ The IC_50_ value of isatin was determined in methanol by Gudipati et al. [24]. The IC_50_ values of stobadine·2HCl ^b^, SME1i-ProC2·HCl ^c^ and SM1M3EC2·HCl ^d^ were determined by Valachova et al. (2014) [23].

## Abbreviations

ABTS	2,2′-Azino-bis(3-ethylbenzothiazoline-6-sulfonic acid) diammonium salt
DPP^•^	2,2-Diphenyl-1-picrylhydrazyl radical
HA	Hyaluronan
ROS	Reactive oxygen species
